# The Influence of Parkinson's Disease Motor Symptom Asymmetry on Hand Performance: An Examination of the Grooved Pegboard Task

**DOI:** 10.1155/2015/307474

**Published:** 2015-11-26

**Authors:** Sara M. Scharoun, Pamela J. Bryden, Michael D. Sage, Quincy J. Almeida, Eric A. Roy

**Affiliations:** ^1^Department of Kinesiology, University of Waterloo, 200 University Avenue West, Waterloo, ON, Canada N2L 3G1; ^2^Department of Kinesiology & Physical Education, Wilfrid Laurier University, 75 University Avenue West, Waterloo, ON, Canada N2L 3C5; ^3^Rehabilitation Sciences Institute, University of Toronto, 500 University Avenue, Toronto, ON, Canada M5G 1V7; ^4^Sun Life Financial Movement Disorders Research and Rehabilitation Centre (MDRC), Wilfrid Laurier University, 66 Hickory Street, Waterloo, ON, Canada N2L 3J5

## Abstract

This study examined the influence of motor symptom asymmetry in Parkinson's disease (PD) on Grooved Pegboard (GP) performance in right-handed participants. The Unified Parkinson's Disease Rating Scale was used to assess motor symptoms and separate participants with PD into two groups (right-arm affected, left-arm affected) for comparison with a group of healthy older adults. Participants completed the place and replace GP tasks two times with both hands. Laterality quotients were computed to quantify performance differences between the two hands. Comparisons among the three groups indicated that when the nonpreferred hand is affected by PD motor symptoms, superior preferred hand performance (as seen in healthy older adults) is further exaggerated in tasks that require precision (i.e., place task). Regardless of the task, when the preferred hand is affected, there is an evident shift to superior left-hand performance, which may inevitably manifest as a switch in hand preference. Results add to the discussion of the relationship between handedness and motor symptom asymmetry in PD.

## 1. Introduction

Parkinson's disease (PD) is defined by asymmetric motor symptoms of tremor, rigidity, and bradykinesia [1, 2]. This right-left asymmetry has been an influential factor when differentiating PD from other, similar movement disorders [3]. The reason for this symptom asymmetry is unknown; however, it may be influenced by handedness [4]. The functional implications of handedness and motor symptom asymmetry are not well understood. However, Munhoz and colleagues [5] recently reported that early onset, left-handedness, and left-sided onset are related to long (≥20 years) ambulatory disease survival, therefore highlighting the importance of understanding the link between handedness and PD motor symptom asymmetry. 

For approximately forty years, scientific debate has existed surrounding whether the preferred limb is initially vulnerable in PD [6]. Acknowledging that a dearth of literature exists analyzing the relationship between functional laterality as reflected in relative hand performance on tests of motor function and asymmetry of motor symptoms associated with the disorder, recent publications have argued whether handedness can be used to predict side of onset of PD. Based on the hand used for writing [7], clinician ratings of handwriting [8], self-reported data for multiple unilateral activities [9], self-reported dominance [10, 11], and documented clinical examination [12], the relationship is still under discussion. A recent systematic review and meta-analysis concluded that a notable association between handedness and lateralization of PD symptoms exists, such that symptom onset is most common in the preferred hand, regardless of hand preference [13]. Furthermore, significant changes of premorbid hand preference have been reported. More specifically, Štochl et al. observed a significant change from right- to left-hand preference when PD onset was on the right and vice versa [14].

Although a relationship has been established, previous studies are limited in their scope of assessing handedness, using hand preference (questionnaires and self-report) as an exclusive means of measurement. Handedness is a multidimensional trait defined by performance and preference [15, 16]. Therefore, it is necessary to consider both dimensions when examining handedness, motor symptom asymmetry, and functional impairments experienced by individuals with PD in everyday life, as assessed in the context of motor tasks.

Observing measures of performance in relation to measures of hand preference, Brown and colleagues [15] identified the place phase of the Grooved Pegboard (GP) test as the best performance-based measure in relation to the Waterloo Handedness Questionnaire as a measure of preference. As an integral component of various neuropsychological assessment batteries, the GP is a standardized, objective measure used to quantify motor functioning of the upper limbs by means of motor speed [17]. Additionally, the GP has two phases, which represent functional aspects of upper limb control including visuomotor ability, fine motor control (place phase), and motor speed (replace phase). To date, the GP has been used as a biomarker of PD associated nigrostriatal denervation [18] and for diagnosing PD in mild-moderate severity individuals [19]. Thus, the GP is an effective, objective tool to investigate the intricacies of handedness and upper limb motor symptoms of PD.

Sage and colleagues [20] used the GP test to assess upper limb motor symptom deficits in PD. However, the complex interaction between handedness and PD symptom asymmetry was beyond the scope of the paper. As such, the current study aimed to further delineate the interaction between PD upper limb motor symptoms and handedness, using the GP as a measure of hand performance in a group of participants with right-hand preference.

To delineate whether differences in performance were a result of age or PD motor symptoms, a group of healthy older adults was included as a control. Although not to the same extent as observed in PD, the hand-arm system is altered in healthy aging. Decreases in muscle mass and strength have been shown to lead to slowness, increased variability in movement, and difficulties with coordination [21]. Studies assessing GP performance in older adults have reported an increase in manual asymmetries, such that older adults take longer to perform with the nonpreferred hand [22, 23]. Based on these observations, it was hypothesized that PD symptoms affecting the preferred arm would lead to the most significant deficits in hand performance and thus lead to a shift towards superior performance with the nonpreferred hand.

## 2. Methods

### 2.1. Participants

This study included 170 right-handed (107 male, 63 female; M_age_ = 71.36, SD = 9.27) participants with PD and 103 right-handed (44 male, 59 female) healthy older adults (ages 60+, exact ages were not recorded for all participants). The institution research ethics board approved the study and all participants signed informed consent before enrolling in the study.

### 2.2. Procedures

While at peak anti-Parkinsonian medication levels, motor symptoms were assessed in participants with PD using the Unified Parkinson's Disease Rating Scale (UPDRS III). The GP test was used as a performance-based measure of hand preference, to assess upper limb function. This included both place and replace components of the task. Hand preference was confirmed by self-report.

The UPDRS [24], administered by a certified and trained evaluator, is a highly used tool for diagnosing and evaluating PD, where the motor examination analyzes motor symptoms and subsequent impairments. Components are scored based on symptom severity from zero to four, where higher scores indicate more severe motor disability. UPDRS items 20–26, which have side-related components, were extracted from the motor examination and used to identify the primary side of the body affected by PD (defined by the absolute difference between right- and left-sided clinical signs) [8, 10]. Those participants with a score of 0 (*n* = 5, 4 male, 1 female) indicative of equivalent symptoms on each side were excluded from analyses. Although inclusion of these participants may be clinically relevant and likely warrants further investigation, assessment was beyond the scope of the current investigation (see Supplementary Material available online at http://dx.doi.org/10.1155/2015/307474 for analysis including these participants). See Table 1 for a summary of demographics for participants included in analyses. 

Following GP (Lafayette Instruments, Lafayette, IN) procedures outlined by Bryden and Roy [17], participants were instructed to move 25 identical 2.5 cm long key-shaped pegs, individually, from a circular receptacle to key-shaped holes of varying orientation on a five by five grid, in a place task. In addition, participants were instructed to complete a modified version of the GP task, requiring them to remove the pegs from the key-shaped holes and return them to the receptacle, in a replace task, suggested to be a purer measure of motor speed [17]. Participants completed two trials of the place and replace task with both the right and left hand, where time to completion was recorded. If participants were unable to complete the GP within five minutes, the trial was ended and a time of 300 seconds was assigned and a second trial was not completed with that limb (*n* = 24 participants with PD). Missing data points resulting from the inability to test a limb during the GP task were coded as missing.

To evaluate asymmetry of GP performance, a laterality quotient was computed for both GP tasks by taking the difference between the left and right hands as a function of the overall movement time of the two hands (L − R/L + R) and multiplying the result by 100. Thus, a positive laterality quotient is indicative of superior performance (i.e., shorter movement time) with the right hand, and a negative laterality quotient is indicative of superior performance with the left hand. Subsequently, the absolute value of the laterality quotient corresponds to the magnitude of the performance difference between the hands.

### 2.3. Data Analyses

SPSS statistical software was used. A mixed analysis of variance test with repeated measures was used to assess laterality quotients computed from the GP task. The between subjects factor was group (healthy older adults: H-OAs; right-side affected PD: RSA-PD; and left-side affected PD: LSA-PD) and the within subjects factor was task (place, replace).

## 3. Results

**Figure 1:**
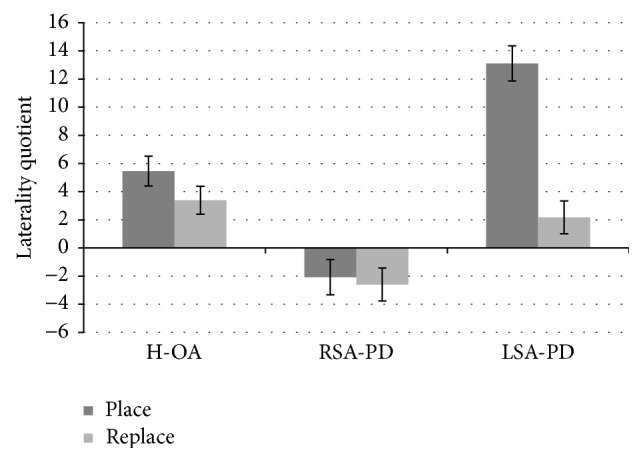
Laterality quotients computed from the GP revealed distinct differences between RSA-PD and LSA-PD groups compared to H-OAs. Error bars represent standard error.

Analyses revealed a main effect of task (*F*(1,248) = 26.06, *p* < .001, *η* ^2^ = .095), such that there were greater laterality quotients (i.e., greater performance differences between the two hands) in place (M = 5.49, SD = 11.83) compared to replace (M = 0.98, SD = 9.97). Additionally, a main effect of group (*F*(2,248) = 32.256, *p* < .001, *η* ^2^ = .206) revealed laterality quotients of all three groups differed. Both H-OA (M = 4.43, SD = 6.78) and LSA-PD (M = 7.64, SD = 13.33) groups had positive laterality quotients, indicative of superior right-hand performance; however, laterality quotients for the LSA-PD group were significantly greater than the H-OAs (*p* = .021). The RSA-PD (M = −2.34, SD = 12.59) had a negative laterality quotient, indicative of superior left-hand performance. Finally, a significant interaction between group and task (*F*(2,248) = 12.591, *p* < .001, *η* ^2^ = .092; see Figure 1) revealed that the laterality quotients of H-OA and LSA-PD groups did not differ in the replace task; however, both groups differed from the RSA-PD group in the place task. All three groups differed in the place task. When comparing the two tasks, RSA-PD and H-OAs had similar laterality quotients in place and replace tasks; however, the LSA-PD group had significantly different laterality quotients.

## 4. Discussion

While PD symptom asymmetry is well known and even used to differentiate PD from other disorders, there is much still not understood, including the functional implications of handedness and symptom asymmetry. Thus, the aim of the current paper was to further delineate the interaction between PD upper limb motor symptom asymmetry and handedness by means of examining hand performance in the GP in a group of right-handed participants.

The current study identified 81 participants with PD as right-side affected and 89 as left-side affected using the UPDRS. Of these participants, 32% met the criteria for asymmetric disease, where 34.5% (*n* = 57) had a right versus left difference score of PD symptoms of at least 5 points, and 8.5% (*n* = 14) had a score of at least 10 points. These proportions are less than reports by Uitti and colleagues, where 46% of their participants had a right versus left difference score of at least 5 points and 12% had a difference score of at least 10 points [8]. Nevertheless, Uitti and colleagues [8] did not find a relationship between the overall magnitude of asymmetric disease and handedness. Left-handed participants in their study did experience increased symptom severity on the left side of the body; however, considering the current work was limited to right-handed participants, variations in results are likely attributed to differences in left- and right-handed individuals with PD.

The division of participants with PD in the current study based on the affected side enabled the comparison of asymmetries in hand performance as a function of PD motor symptom asymmetry. As seen in Figure 1, evident differences emerged when comparing laterality quotients in the two groups of participants with PD and a group of healthy older adults. We will begin with discussion of the replace task, as this requires less motor skill and thus represents a measure of motor speed. Here, H-OA and LSA-PD groups did not differ; however, both groups displayed significantly different laterality quotients than the RSA-PD group. Put simply, H-OAs demonstrated a positive laterality quotient, indicative of superior right-hand performance, as expected based on previous findings [22, 23]. The same was true for the LSA-PD group, which indicates that when the nonpreferred side of right-handed individuals is affected by PD motor symptoms, there is no evident difference in motor speed, in this context. That said both of the aforementioned groups differed from the RSA-PD group, where their laterality quotient was negative and thus indicative of superior left-hand performance. This extends the work by Štochl and colleagues [14] who noted a significant change from right- to left-hand preference when PD onset was on the right and vice versa. Štochl and colleagues' [14] methods were limited to a 7-item hand preference questionnaire, where premorbid hand preference was collected retrospectively; therefore, the current study provides additional support that PD motor symptom asymmetry does indeed influence handedness of individuals with PD.

Next, we will address results of the place task, which requires an increase in fine motor precision compared to the replace task. As such, the place task can be considered a better gauge of functional ability of the upper limb, as it requires more fine motor skill. The laterality quotients of all three groups differed in the place task. Similar to the replace task, the RSA-PD group demonstrated negative laterality quotients, indicating a shift to superior left-hand performance, whereas both H-OA and LSA-PD groups displayed positive laterality quotient, indicative of superior right-hand performance. Unlike the replace task, the LSA-PD group's laterality quotient was significantly more positive than the H-OA group. Furthermore, when comparing the two tasks, the laterality quotients of both H-OA and RSA-PD groups did not differ; however, the LSA-PD group's laterality quotient was significantly more positive in the place task.

Summarizing, results of the current study indicate that when the nonpreferred hand of right handers is affected by PD motor symptoms, superior preferred hand performance seen in H-OAs [22, 23] is further exaggerated in tasks that require precision. Regardless of whether the task requires precision, or simply speed, when the preferred hand of right handers is affected, there is an evident shift to superior left-hand performance, which may inevitably manifest as a switch in hand preference [14].

Previous reports indicate that the preferred side is primarily affected at the onset of PD symptoms [7]. In fact, Barrett and colleagues [10] have noted that handedness plays a role in the “reported first symptom, the time to diagnosis and the time to dopaminergic treatment initiation” (p. 1122). In light of the current findings, which indicate the most debilitating effects on hand performance when the preferred side is affected, this research has implications for the identification and management of PD. Nevertheless, it is important to consider that patients affected on their preferred side are more likely to report upper limb dysfunction prior to those affected on their nonpreferred side (who generally first report impairments in their lower limbs) [10, 11]. As such, symptoms may have actually first appeared on the nonpreferred side; however, they were not recognized until the preferred side was also affected.

One question of interest here is whether the motor symptoms of PD which give rise to PD initially in the preferred hand are the same as those seen when first expressed in the nonpreferred hand. A difference in symptoms underlying these two expressions of PD (i.e., preferred versus nonpreferred hand first affected) may have implications for how rapidly the disease progresses and whether the motor symptoms are accompanied by other disorders such as in cognition. This question is beyond the scope of this paper but has been addressed in some of our other works (e.g., [20]).

## 5. Conclusions

Taken together, our findings help clarify the complex interactions of handedness, motor symptom asymmetry, and progression on functional upper limb control. Overall, the observed results are similar to those recently discussed in the literature, noting the obvious effect of the disease on functional preferred hand use, as was demonstrated by poor performance on the GP task when the preferred hand was affected by PD. Results are clinically relevant, as they aid in our understanding of disease progression. Future studies should aim to include a comparable sample of right- and left-handed participants to examine similarities and differences in performance. Additionally, a handedness inventory, such as the Waterloo Handedness Questionnaire [25], should be incorporated to enable comparison between participants based on the degree (i.e., strength) of handedness, as opposed to simply direction (i.e., left or right).

## Supplementary Material

Participants with equivalent symptoms on each side (n = 5, 4 male, 1 female) were excluded from analyses. Although inclusion of these participants was beyond the scope of the current investigation, analyses including them can be found in the supplementary material.

## Figures and Tables

**Table 1:** Demographic characteristics of participants with PD.

	Right-side affected	Left-side affected
Number	79	86
Male : female	49 : 30	54 : 32
Age	M = 70.21, SD = 10.36	M = 71.78, SD = 8.32
UPDRS	M = 27.46, SD = 10.30	M = 29.68, SD = 10.71
Right UPDRS	M = 11.67, SD = 3.86	M = 7.92, SD = 3.83
Left UPDRS	M = 7.14, SD = 4.06	M = 11.99, SD = 4.11
|Right–left|	M = 5.13, SD = 3.25	M = 4.28, SD = 3.02
*0.5*–*4.5*	*n *= 41	*n *= 53
*5*	*n *= 28	*n *= 29
*10+*	*n *=10	*n *= 4

## References

[1] Djaldetti R., Ziv I., Melamed E. (2006). The mystery of motor asymmetry in Parkinson's disease. *Lancet Neurology*.

[2] Jankovic J. (2008). Parkinson's disease: clinical features and diagnosis. *Journal of Neurology, Neurosurgery & Psychiatry*.

[3] Hughes A. J., Daniel S. E., Kilford L., Lees A. J. (1992). Accuracy of clinical diagnosis of idiopathic Parkinson's disease: a clinico-pathological study of 100 cases. *Journal of Neurology Neurosurgery & Psychiatry*.

[4] Melamed E., Poewe W. (2012). Taking sides: is handedness involved in motor asymmetry of Parkinson's disease?. *Movement Disorders*.

[5] Munhoz R. P., Espay A. J., Morgante F. (2013). Long-duration Parkinson's disease: role of lateralization of motor features. *Parkinsonism & Related Disorders*.

[6] Reynolds L. M., Locke S. (1971). Relation between handedness and side of onset of Parkinsonism. *The Lancet*.

[7] Yust-Katz S., Tesler D., Treves T. A., Melamed E., Djaldetti R. (2008). Handedness as a predictor of side of onset of Parkinson's disease. *Parkinsonism & Related Disorders*.

[8] Uitti R. J., Baba Y., Whaley N. R., Wszolek Z. K., Putzke J. D. (2005). Parkinson disease: handedness predicts asymmetry. *Neurology*.

[9] Štochl J., Hagtvet K. A., Brožová H., Klempíř J., Roth J., Růžička E. (2009). Handedness does not predict side of onset of motor symptoms in Parkinson's disease. *Movement Disorders*.

[10] Barrett M. J., Wylie S. A., Harrison M. B., Wooten G. F. (2011). Handedness and motor symptom asymmetry in Parkinson's disease. *Journal of Neurology, Neurosurgery and Psychiatry*.

[11] Stewart K. C., Fernandez H. H., Okun M. S., Rodriguez R. L., Jacobson C. E., Hass C. J. (2009). Side onset influences motor impairments in Parkinson disease. *Parkinsonism & Related Disorders*.

[12] van der Hoorn A., Bartels A. L., Leenders K. L., de Jong B. M. (2011). Handedness and dominant side of symptoms in Parkinson's disease. *Parkinsonism and Related Disorders*.

[13] van der Hoorn A., Burger H., Leenders K. L., de Jong B. M. (2012). Handedness correlates with the dominant parkinson side: a systematic review and meta-analysis. *Movement Disorders*.

[14] Štochl J., Croudace T. J., Brožová H., Klempíř J., Roth J., Růžička E. (2012). Changes of hand preference in Parkinson's disease. *Journal of Neural Transmission*.

[15] Brown S. G., Roy E. A., Rohr L. E., Snider B. R., Bryden P. J. (2004). Preference and performance measures of handedness. *Brain and Cognition*.

[16] Corey D. M., Hurley M. M., Foundas A. L. (2001). Right and left handedness defined: a multivariate approach using hand preference and hand performance measures. *Neuropsychiatry, Neuropsychology and Behavioral Neurology*.

[17] Bryden P. J., Roy E. A. (2005). A new method of administering the Grooved Pegboard Test: performance as a function of handedness and sex. *Brain and Cognition*.

[18] Bohnen N. I., Kuwabara H., Constantine G. M., Mathis C. A., Moore R. Y. (2007). Grooved pegboard test as a biomarker of nigrostriatal denervation in Parkinson's disease. *Neuroscience Letters*.

[19] Bohnen N. I., Studenski S. A., Constantine G. M., Moore R. Y. (2008). Diagnostic performance of clinical motor and non-motor tests of Parkinson disease: a matched case-control study. *European Journal of Neurology*.

[20] Sage M. D., Bryden P. J., Roy E. A., Almeida Q. J. (2012). The relationship between the grooved pegboard test and clinical motor symptom evaluation across the spectrum of Parkinson's disease severity. *Journal of Parkinson's Disease*.

[21] Seidler R. D., Bernard J. A., Burutolu T. B. (2010). Motor control and aging: links to age-related brain structural, functional, and biochemical effects. *Neuroscience and Biobehavioral Reviews*.

[22] Sivagnanasunderam M., Gonzalez D. A., Bryden P. J., Young G., Forsyth A., Roy E. A. (2015). Handedness throughout the lifespan: cross-sectional view on sex differences as asymmetries change. *Frontiers in Psychology*.

[23] Weller M. P. I., Latimer-Sayer D. T. (1985). Increasing right hand dominance with age on a motor skill task. *Psychological Medicine*.

[24] Fahn S., Elton R. L., Fahn S., Masrden C. D., Calne D., Goldsteing D. (1987). Unified Parkinson's disease rating scale. *Recent Development in Parkinson's Disease*.

[25] Steenhuis R. E., Bryden M. P., Schwartz M., Lawson S. (1990). Reliability of hand preference items and factors. *Journal of Clinical and Experimental Neuropsychology*.

