# Supplementary material for: The Influence of Parkinson's Disease Motor Symptom Asymmetry on Hand Performance: An Examination of the Grooved Pegboard Task
Source: Parkinsons Dis. 2015 Nov 26;2015:307474. doi: 10.1155/2015/307474 (PMC4674610; doi:10.1155/2015/307474)
Supplement: Supplementary file 1 — Participants with equivalent symptoms on each side (n = 5, 4 male, 1 female) were excluded from analyses. Although inclusion of these participants was beyond the scope of the current investigation, analyses including them can be found in the supplementary material. [file 307474.f1.pdf]

Supplementary Material

A mixed analysis of variance test with repeated measures was used to assess laterality quotients computed from the GP task. The between subjects factor was group (healthy older adults: H-OAs; right-side affected PD: RSA-PD; left-side affected PD: LSA-PD; and Bilateral) and the within subjects factor was task (Place, Replace).

Analyses revealed a main effect of task (F (1, 251) = 9.390, p = .002, η^2 = .036), such that there were greater laterality quotients (i.e., greater performance differences between the two hands) in place (*M* = 5.40, *SD* = 13.47) compared to replace (*M* = 1.33, *SD* = 10.28). Additionally, a main effect of group (F (2, 251) = 22.344, p < .001, η^2 = .211) revealed laterality quotients of all three groups differed, with the exception of Bilateral, which did not differ from H-OAs or LSA-PD. Both H-OA (*M* = 4.43, *SD* = 6.78), LSA-PD (*M* = 7.64, *SD* = 13.33) and Bilateral (*M* = 9.862, *SD* = 12.26) groups had positive laterality quotients, indicative of superior right-hand performance; however, laterality quotients for the LSA-PD group were significantly greater than the H-OAs (p = .021). The RSA-PD (*M* = -2.34, *SD* = 12.59) had a negative laterality quotient, indicative of superior left-hand performance.

Finally, a significant interaction between group and task (F (2, 51) = 8.60, p < .001, η^2 = .093; see Figure 1) revealed that the laterality quotients of H-OA, LSA-PD and Bilateral groups did not differ in the replace task; however, H-OA and LSA-PD group differed from the RSA-PD group in the place task. All three groups differed in the place task. When comparing the two tasks, RSA-PD, Bilateral and H-OA groups had similar laterality quotients in place and replace tasks; however, the LSA-PD group had significantly different laterality quotients.

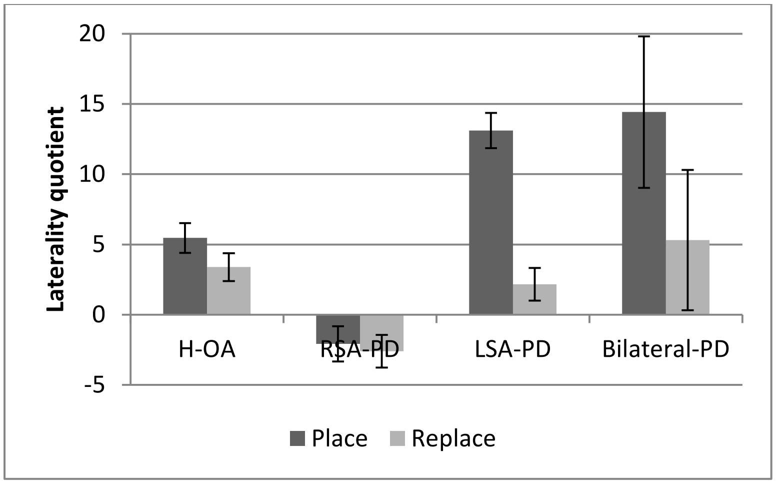

Supplementary material figure 1: Laterality quotients computed from the GP revealed distinct differences between RSA-PD and LSA-PD groups compared to H-OAs and Bilateral-PD. Error bars represent standard error.
